# Predictive Value of Diffusion, Glucose Metabolism Parameters of PET/MR in Patients With Head and Neck Squamous Cell Carcinoma Treated With Chemoradiotherapy

**DOI:** 10.3389/fonc.2020.01484

**Published:** 2020-09-02

**Authors:** András Kedves, Zoltán Tóth, Miklós Emri, Krisztián Fábián, Dávid Sipos, Omar Freihat, József Tollár, Zsolt Cselik, Ferenc Lakosi, Gábor Bajzik, Imre Repa, Árpád Kovács

**Affiliations:** ^1^Doctoral School of Health Sciences, University of Pécs, Pécs, Hungary; ^2^Department of Medical Imaging, Faculty of Health Sciences, University of Pécs, Pécs, Hungary; ^3^Dr. József Baka Diagnostic, Radiation Oncology, Research and Teaching Center, “Moritz Kaposi” Teaching Hospital, Kaposvár, Hungary; ^4^MEDICOPUS Healthcare Provider and Public Nonprofit Ltd., Somogy County Moritz Kaposi Teaching Hospital, Kaposvár, Hungary; ^5^Department of Medical Imaging, Faculty of Health Sciences, University of Debrecen, Debrecen, Hungary; ^6^Oncoradiology, Csolnoky Ferenc County Hospital, Veszprém, Hungary; ^7^Department of Oncoradiology, Faculty of Medicine, University of Debrecen, Debrecen, Hungary

**Keywords:** PET/MR, predictive value, SUV, MTV, TLG, ADC, clinical outcome, squamous cell carcinoma

## Abstract

**Purpose:** This study aims to evaluate the predictive value of the pretreatment, metabolic, and diffusion parameters of a primary tumor assessed with PET/MR on patient clinical outcomes.

**Methods:** Retrospective evaluation was performed using PET/MR image data sets acquired using the single tracer injection dual imaging of 68 histologically proven head and neck cancer patients 4 weeks before receiving definitive chemoradiotherapy (CRT). PET/MR was performed before the CRT and 12 weeks after the CRT for response evaluation. Image data (PET and MRI diffusion-weighted imaging [DWI]) was used to specify the maximum standard uptake value, the peak lean body mass corrected, SUV_max_, the metabolic tumor volume, the total lesion glycolysis (SUV_max_, SUL_peak_, MTV, and TLG), and the mean apparent diffusion coefficient (ADC_mean_) of the primary tumor. Based on the results of the therapeutic response evaluation, two patient subgroups were created: one with a viable tumor and another without. Metabolic and diffusion data, from the pretreatment PET/MR and the therapeutic response, were correlated using Spearman's correlation coefficient and Wilcoxon's test.

**Results:** After completing the CRT, a viable residual tumor was detected in 36/68 (53%) cases, and 32/68 (47%) patients showed complete remission. However, no significant correlation was found between the pretreatment parameter, ADC_mean_ (*p* = 0.88), and the therapeutic success. The PET parameters, SUV_max_ and SUL_peak_, MTV, and TLG (*p* = 0.032, *p* = 0.01, *p* < 0.0001, *p* = 0.0004) were statistically significantly different between the two patient subgroups.

**Conclusion:** This study found that MRI-based (ADC_mean_) data from FDG PET/MR pretreatment could not be used to predict therapeutic response although the PET parameters SUV_max_, SUL_peak_, MTV, and TLG proved to be more useful; thus, their inclusion in risk stratification may also be of additional value.

## Key Points

**Question:** How can ^18^F-FDG PET/MR values predict the prognosis of head and neck cancer before treatment?

**Pertinent findings:** The retrospective study reveals correlations between baseline single ^18^F-FDG tracer injection dual imaging acquisition PET-based parameters (SUV_max_, SUL_peak_, MTV, and TLG) and MR DWI (ADC_mean_)-based parameter and therapy response after treatment (CR, NCR).

**Implications for patient care:** Clinicians should measure and integrate the suggested parameters (SUV_max_, SUL_peak_ MTV, and TLG) with PET/MR to provide the most accurate therapy for the patient.

## Introduction

Head and neck carcinomas are the sixth most common cancers nowadays. These carcinomas make up 6% of all new cancer cases recorded yearly (1, 2). The majority of head and neck carcinomas belong to the histopathological group of squamous cell carcinoma of the head and neck (HNSCC) (3).

The main clinical staging component for diagnosing HNSCC is the endoscopy, but conventional radiological staging methods, such as computed tomography (CT) and magnetic resonance imaging (MRI) have proven more accurate and informative in setting up a diagnosis (4).

Beyond these conventional imaging methods, hybrid imaging has also shown an outstanding staging ability, particularly in detecting or characterizing head and neck cancers (5). Hybrid imaging, such as positron emission tomography/computed tomography (PET/CT) or PET/MR, is an imaging solution that could be used simultaneously with anatomical information to provide metabolic data (with a ^18^F-FDG: 2-Deoxy-2-[18F]fluoro-D-glucose [^18^F-FDG] tracer). With the information obtained using hybrid imaging, oncological practice could conduct many diagnostic or therapeutic procedures, for example, whole-body staging/restaging, irradiation planning, or even the evaluation of the disease prognosis (6, 7).

To characterize HNSCC, it is essential to use PET imaging with an ^18^F-FDG tracer. Multiparametric data obtained from the ^18^F-FDG PET evaluation was not only linked with histopathologically confirmed tumor properties, but also connected with PET/MR parameters (such as the apparent diffusion coefficient, ADC, derived from diffusion-weighted imaging [DWI] examinations, the maximum standardized uptake value [SUV_max_], and the peak lean body mass corrected, SUV_max_ [SUL_peak_]), and treatment-associated failure, locoregional recurrence, and death (8). In many malignancies, ^18^F-FDG accumulation (in the most common form, known as SUV) appears to be a good indicator of disease aggressiveness (9). There are numerous studies aimed at the utility of FDG PET parameters in predicting response to CRT in head and neck cancer specifically (10, 11).

The PET/MR method, combined with conventional contrast-enhanced (CE) MR sequences, is excellent for getting data on anatomical and metabolic conditions although data on the cellular diffusion of the scanned area could also be derived via DWI methods during the MR acquisition (12). Besides the clinical and histopathological factors, imaging parameters may provide important prognostic biomarkers in different malignancies (8). DWI can measure water molecules' movement and the tumor cell density in tissue *in vivo* (13). DWI methods could be used for staging HNSCC. In some cases, DWI allows for a more accurate staging method than PET/CT (e.g., evaluating cN0) (14). DWI-derived variables, such as ADC, may have a prognostic and predictive value that relate to the post-therapeutic status of the disease and the outcome of chemoradiotherapy (15). To investigate the predictive value of ADC, scans must be performed before and after treatment. According to the results using this method, ADC could be an indicator of locoregional failure, which is a component of the treatment response. The ADC mean values (ADC_mean_) are, therefore, possible parameters for prediction, per the suggestion of Martens et al. (16). Leifels et al. found that tumor metabolism, cellularity, and perfusion show complex relationships in HNSCC. Furthermore, these associations depend on tumor grading (17).

Moreover, metabolic tumor volume (MTV) and total lesion glycolysis (TLG) seem to be predictors of the postoperative survival of patients that have been diagnosed *via* PET/CT with MTV seeming to be a better predictor than TLG (18). Overall, there are numerous studies aimed at the utility of FDG PET parameters in predicting response to CRT in head and neck cancer specifically (10, 11).

This study aimed to determine the best predictors for the treatment outcome of patients diagnosed using a single tracer injection dual imaging acquisition protocol in PET/MR from a set of previously described parameters (SUV_max_, SUL_peak_, ADC_mean_, MTV, and TLG). This study also aimed to evaluate the connection between the possible above parameters that prove to be the most predictive of the HNSCC outcome.

## Materials and Methods

### Patients and Treatment

**Figure 1:**
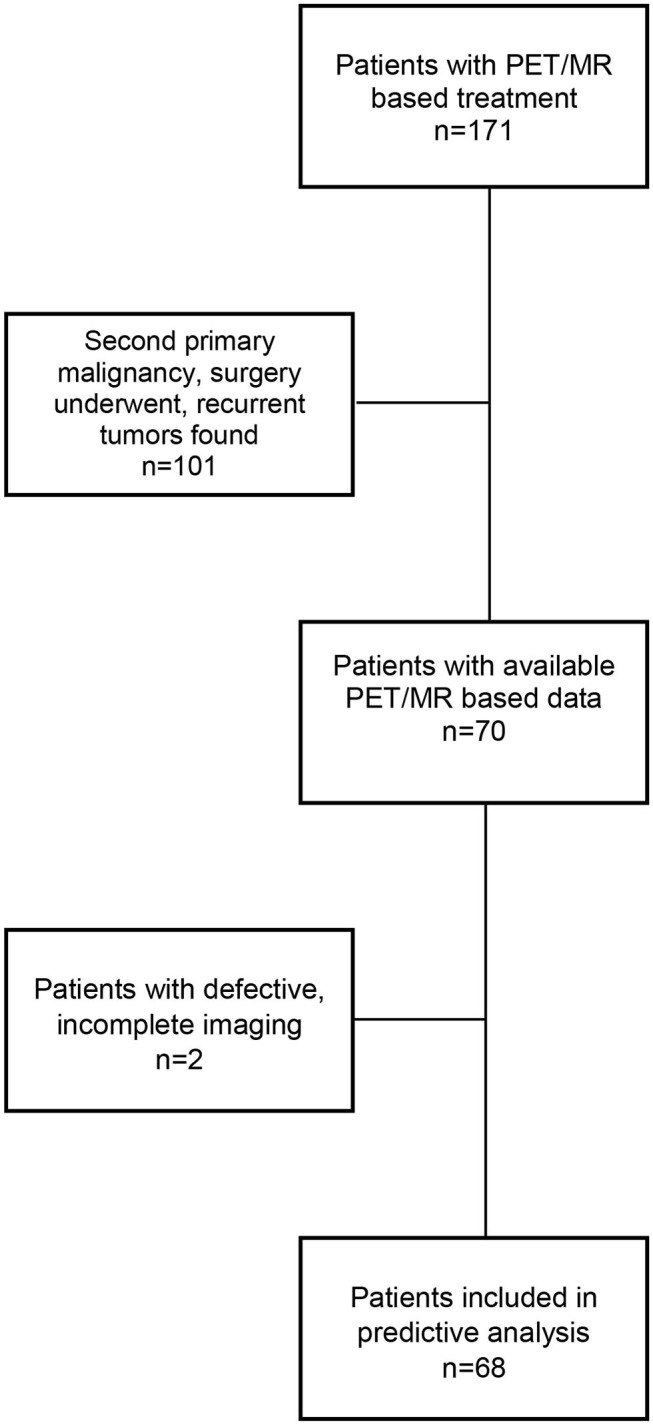
Flowchart of excluded patients.

Informed consent was waived by the local ethics committee and the institutional review board (IRB). Between October 2015 and May 2019, 68 pathologically confirmed, HNSCC patients (male:female ratio of 3:1) with a median age of 61 ± 8 years (range, 46–87) were enrolled in the current retrospective study. All patients underwent 3-D-fused ^18^F-FDG PET/CT volumetric modulated arc therapy (VMAT)–based, definitive image-guided irradiation (IGRT, with a daily cone-beam CT) and concomitant chemotherapy (with 40 mg/ml cisplatin protocol weekly) up to 70 Gy in the Dr. József Baka Diagnostic, Radiation Oncology, Research, and Teaching Center, “Moritz Kaposi” Teaching Hospital, Kaposvár, Hungary. Exclusion criteria were (1) patients with second primary malignancy, (2) patients with previous history of surgery, and (3) patients with recurrent primary tumors (Figure 1).

All patients underwent pretreatment staging (during the planning process 4 weeks before treatment) and post-treatment (12 weeks after treatment) PET/CT and PET/MR for a short-term follow-up. Per the eighth edition of the Union for International Cancer Control (UICC) TNM Project 8th TNM staging system, 5/68 (8%) patients had T1 disease, 21 (31%) patients had T2 disease, 23 (33%) patients had T3 disease, and 19 (28%) patients had T4 disease. Meanwhile, 33 of the patients had a histopathologically confirmed (supported by ultrasound [US] guided biopsy) locoregional lymph node while 35 showed an absence of metastatic lymphoid glands. Grade distribution were as follow: G1 (*n* = 15), G2 (*n* = 35), and G3 (*n* = 18). *N* category was as follows: N0 (*n* = 35), N1 (*n* = 19), N2 (*n* = 9), and N3 (*n* = 5).

Primary tumor localizations were: pharyngeal (*n* = 32), sublocalized into seven patients nasopharyngeal, 13 patients oropharyngeal, and 12 patients with hypopharyngeal. Laryngeal (*n* = 36), sublocalized into 26 with supraglottic, four glottic, and six subglottic. The epidemiology data specific to the tumor and lymph node and the response to therapy are summarized in Table 1.

**Table 1:** Values are presented as the number of patients (%) unless otherwise indicated.

**Characteristics**	**Value (%)**
Number of patients	68 (100)
Mean age (year)	61 ± 8 (46–87)
**Sex**	
Men	44 (65)
Women	24 (35)
**Localization**	
Pharynx	
Epipharynx	7 (10)
Mesopharynx	13 (19)
Hypopharynx	12 (18)
**Larynx**	
Supraglottic	26 (38)
Glottic	4 (6)
Subglottic	6 (9)
**Treatment Response Groups**	
Complete remission	36 (53)
Partial remission	16 (24)
Stable disease	9 (13)
Progressive disease	7 (10)
**Treatment Response Related Groups**	
CR	36 (53)
non-CR	32 (47)
**Initial T Stage**	
I.	5 (8)
II.	21 (31)
III.	23 (33)
IV.	19 (28)
**Grade**	
I.	15 (23)
II.	35 (51)
III.	18 (26)
**Presence of Lymph Node**	
Yes	33 (49)
No	35 (51)
**Lymph Node Stage**	
0	35 (52)
1	19 (28)
2	9 (13)
3	5 (7)

### PET/MR Acquisition

Examinations were performed using a hybrid PET/MR scanner (Biograph mMR, Siemens Healthcare GmbH, Erlangen, Germany). Blood glucose level was checked before tracer injection to ensure the patients were euglycemic. The patients received intravenous administration of 4 MBq/kg activity of ^18^F-FDG. Then, PET/CT (Truepoint 64, Siemens Healthcare GmbH, Erlangen, Germany) was performed, using FDG initially injected for PET/CT (60 ± 10 min of the uptake period) before PET/MR (15 ± 5 min after PET/CT). Further tracer injection was not applied for PET/MR (single tracer injection dual imaging acquisition protocol). After proper patient preparation (removal of metal implants, hearing aids, metal objects in the region), images were obtained of the head and neck position using dedicated coils. Thus, only PET/MR parameters were included in the research.

Native MRI sequences were T2-weighted TSE turbo inversion recovery magnitude (TIRM) (TR/TE/TI 3300/37/220 ms, FOV: 240 mm, slice thickness: 3 mm, 224 × 320) coronal and T1-weighted turbo spin-echo (TSE) (TR/TE 800/12 ms, FOV: 200 mm, slice thickness: 4 mm, 224 × 320), and T1-weighted TSE Dixon fat suppression (FS) (TR/TE 6500/85 ms, FOV: 200 mm, slice thickness: 4 mm, 256 × 320) transversal and acquired without an intravenous contrast agent.

**Figure 2:**
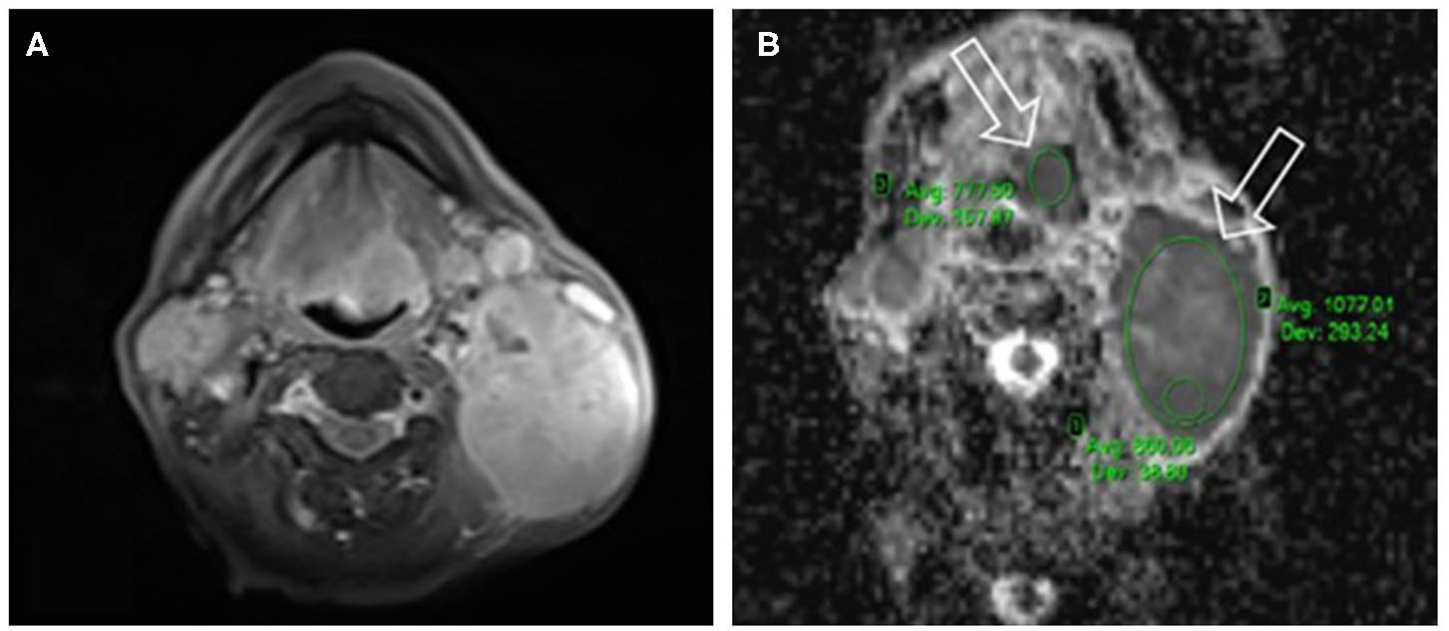
Axial Dixon fat suppressing (FS) T1-weighted turbo spin echo (TSE) sequence **(A)** used as an anatomical map. On the corresponding apparent diffusion coefficient (ADC)-map **(B)** the area of low signal intensity, epiglottic primary tumor's (left white arrow) mean ADC (ADC_mean_: 777.5 + -42.2 10^−6^ s/mm^2^) delineated (with left green ellipse), and the metastasis of largest lymph node (right white arrow) on the left side of the neck.

Diffusion-weighted (DW) measurement was done as part of a routine examination. In this case, a 2-D spin-echo DWI echo-planar (EP) sequence (FOV: 315 mm, TR: 9,900 ms, TE minimum: 70 ms, TI 200 ms, slice thickness: 5 mm) was used. An ADC map was automatically generated from the DWI pictures via the implemented software. The restricted diffusion rate was quantified by calculating the apparent diffusion coefficient. To reduce the perfusion effect (on the ADC calculation, a 50 s/mm^2^ “b” value was used as the first measurement (the other *b* values were 800 and 1,000 s/mm^2^). Furthermore, an axial Dixon FS T1-weighted TSE sequence and a coronal TSE Dixon FS sequence were conducted after 0.1 mmol per kg of bodyweight contrast material (Gadovist Bayer Healthcare, Leverkusen, Germany) was injected into the patient (Figure 2). The imaging was repeated after the completion of the CRT for therapeutic response assessment.

For PET data collection, a magnetic resonance–based attenuation correction ([MRAC], using a CAIPIRINHA-accelerated T1-weighted Dixon 3D-VIBE sequence) was used for PET attenuation correction, and the wide range bed position PET Emission scan was acquired for 900 s with a fixed FOV range (20 cm) and a (172 × 172) matrix without bed movement. An iterative ordered subset expectation maximization (3-D OP-OSEM) PET image reconstruction algorithm was used with three iterations and eight subsets as well as 4-mm Gaussian filtering settings. PET data was corrected for scatter, random coincidences, and attenuation using the MR data.

### Image Analysis

Metabolic parameters were calculated using a dedicated Syngo.via (Siemens Medical Solutions, VB20, Siemens Healthcare GmbH, Erlangen, Germany) multimodality image evaluation and postprocessing application based on fusioned PET/MR imaging. The SUV_max_, SUL_peak_, MTV, and TLG data of the primary head and neck cancers were collected using the volume of interest (VOI) technique. This study was built only on one observer assessment. VOIs were assessed by a nuclear medicine physician with 15 years of experience. The SUV_max_ represents single voxel activity concentration in a particular lesion with the highest uptake. The SUL_peak_ is defined as a lean body mass normalized-average SUV value measured in a 1 cm^3^ volume spheric region of interest (ROI) centered around the hottest point in the tumor foci. For the MTV and TLG definition, the relative threshold at 50% of tumor SUV_max_ was used as proposed by Deron et al. (19) where MTV represents the volume of the above given VOI while TLG is the product of the VOI average SUL (SUL_mean_) multiplied by the corresponding MTV.

The localization of lesions was assessed on the ADC map using eRAD PACS Desktop Viewer 8.0 software. This study applied the single slice measurement method; we have chosen the largest and the most homogeneous part of the tumor as a standard for all objects (20). ROI was placed manually on the most solid part of the tumor, which shows the highest signal intensity on DWI images (hyperintense) and hypointense on ADC map (21, 22). ROIs were measured by a radiologist with 10 years of experience in DWI measurement. Thus, during the ADC measurement, the researchers took precautions, such as excluding areas of gross necrosis from the sample (ROI) while plotting an elliptic ROI (15). In all lesions, ADC_mean_ was used as a standard measurement unit to minimize the effect of tumor heterogeneity, it also was the standard unit to be used as a reliable parameter because it reflects the heterogeneity of the tumor in the specified slice to enable the researcher to distinguish the different entities in the same image (5, 6, 22) (Figure 2).

### Clinical Evaluation

To evaluate the therapeutic tumor responses based on pre- and post-treatment PET/MR and PET/CT information, the European Organization for Research and Treatment of Cancer (EORTC) (23) system was used. Two patient groups were established according to the results of the PET/MR therapeutic response evaluation and the clinical follow-up. Furthermore, patient subgroups were also set up, namely a complete remission (CR) group defined as patients with an absence of a viable primary tumor tissue and a non-complete remission (NCR) group defined as patients with any pernicious proliferations including partial response, stable disease, and progressive disease groups (24, 25).

### Statistical Analysis

For all the statistical analyses conducted, R-scripts developed in-house based on the R-software environment for statistical computing (version 3.3.1; R Foundation for Statistical Computing, Vienna, Austria)^1^ were used together with ggpubr^2^ and summarytools^3^ software packages.

The Shapiro-Wilks test (26) was used to check the normality of the measured SUV_max_, SUL_peak_, TLG, MTV, and ADC_mean_ data. Because these tests showed non-normality distributions of the SUV_max_ (*p* < 0.0001), SUL_peak_ (*p* = 0.0001), TLG (*p* < 0.0001), and MTV (*p* < 0.0001) in the population, Spearman's correlation coefficient was used to describe the strength of the correlation between the data pairs, and Wilcoxon's rank-sum test was used for group comparison. The estimated parameters were correlated in different tumor subgroups (grade 1–2 and 3) per suggestion of Leifels et al. (17).

## Results

A total of 68 patients were enrolled in this study. The patients' characteristics are summarized in Table 1. Well-visualized primary lesions were defined in all patients with the initial ^18^F-FDG PET/MR.

The mean SUV_max_, SUL_peak_, TLG, MTV, and ADC_mean_ (+SD) values measured from the patients' primary tumors were 9.05 ± 6.55 (range, 3.43–41.22), 6.95 ± 5.50 (range, 2.91–32.34), 121.48 ± 163.09 (range, 4.72–570.60), 25.88 ± 21.49 cm^3^ (range, 1.38–110.52), and 933.34 ± 136.15 10^−6^ mm^2^/s (range, 610.29–1337.85), respectively (Table 2).

**Table 2:** Estimated average parameters in the sublocalizations of head and neck.

	**Min**.					**Max**.					**Mean**				
	**SUV_**max**_ (lbm)**	**SUL_**peak**_ (SUV-lbm / Size)**	**TLG** **(SUV-lbm × cm^**3**^)**	**MTV (cm^**3**^)**	**ADC_**mean**_** **10^**−6**^mm/s^**2**^**	**SUV_**max**_ (lbm)**	**SUL_**peak**_(SUV-lbm / Size)**	**TLG** **(SUV-lbm × cm^**3**^)**	**MTV (cm^**3**^)**	**ADC_**mean**_** **10^**−6**^mm/s^**2**^**	**SUV_**max**_ (lbm)**	**SUL_**peak**_(SUV-lbm / Size)**	**TLG (SUV-lbm × cm^**3**^)**	**MTV (cm^**3**^)**	**ADC_**mean**_ 10^**−6**^mm/s^**2**^**
Epipharynx	2.7	2.2	4.0	2.3	640.1± 63	13.8	12.0	502.3	176.7	1212.3± 73	8.15	6.6	217.6	42.4	967.6 ± 68
Mesopharynx	3.1	2.5	3.7	2.5	622.1± 75	9.3	9.3	59.1	13.6	1107.6± 84	8.8	5.1	9.4	5.7	940.4 ± 79
Hypopharynx	3.1	2.9	25.0	8.6	613.1± 69	20.9	18.3	475.5	89.1	1200.4± 65	10.1	8.4	152.6	29.4	955.5 ± 67
Supraglottic	6.2	4.4	12.7	4.5	730.1± 70	10.8	9.2	163.4	37.0	1337.9± 80	8.3	6.8	80.4	15.3	910.1 ± 75
Glottic	5.7	4.3	15.5	4.4	690.1± 63	16.5	13.8	169.6	31.1	1095.5± 79	9.9	7.7	65.7	15.3	908.4 ± 71
Subglottic	5.7	4.1	22.5	7.0	610.3± 61	16.1	12.3	344.3	304.6	1144.6± 79	8.9	7.1	203.9	47.2	920.2 ± 70

### Correlation Analysis

**Figure 3:**
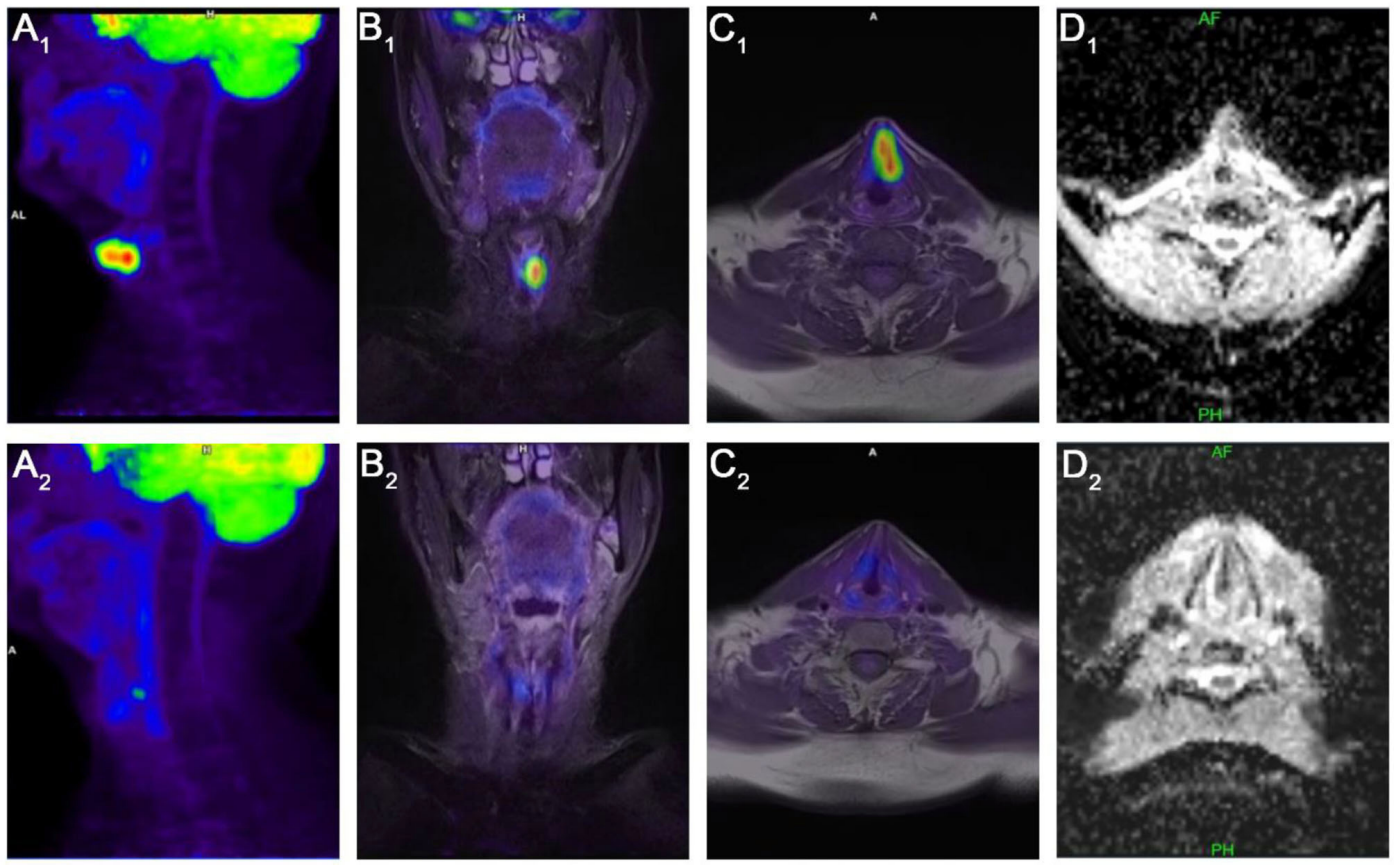
Complete Remission (CR): sagittal PET **(A**_**1**_**)**, coronal **(B**_**1**_**)**, and axial **(C**_**1**_**)** PET/MR images and axial MR-diffusion weighted imaging (DWI) apparent diffusion coefficient (ADC) map of the tumor **(D**_**1**_**)** show the glottic tumor spread over the supra-, and infraglottic regions, pretreatment maximum of standardized uptake value (SUV_max_): 14.12, peak lean body mass corrected SUV_max_ (SUL_peak_): 8.93, total lesion glycolysis (TLG): 25.46, metabolic tumor volume: 2.97 cm^3^, mean ADC (ADC_mean_): 867.22 + -53.52 × 10^−6^ s/mm^2^. Post-treatment sagittal PET **(A**_**2**_**)**, coronal **(B**_**2**_**)**, and PET/MR and DWI ADC axial **(D**_**2**_**)** images show complete remission (CR); without any pathologic FDG accumulation, and diffusion restriction on the observed volume.

Based on the restaging, the PET/MR scans for CR (Figure 3) were achieved for 36/68 (53%) patients while viable tumor was observed in 32/68 (47%) patients.

**Figure 4:**
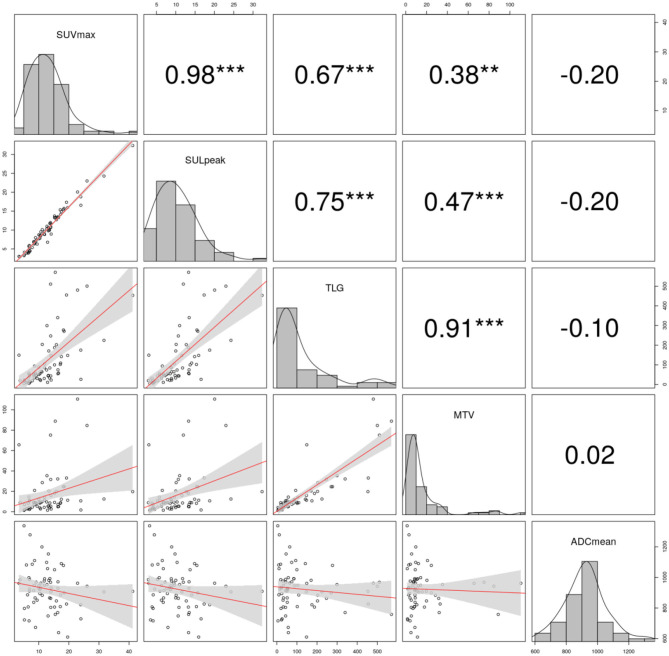
Correlation plots (lower triangle), histograms (diagonal), and Spearman correlation coefficients in the upper triangle are shown in the plot-matrix of pretreatment maximum of standardized uptake value (SUV_max_), peak lean body mass corrected SUV_max_ (SUL_peak_), total lesion glycolysis (TLG), metabolic tumor volume (MTV), mean apparent diffusion coefficient (ADC_mean_). The significance levels are denoted by stars (**p < 0.01, ***p < 0.001, no star: no significant).

The results of the correlation analysis are summarized in Figure 4. No significant correlation between SUV_max_, SUL_peak_, TLG, MTV, and the ADC_mean_ for the patients diagnosed using the single tracer injection dual imaging acquisition protocol was noted.

On the next step, in two separated tumor subgroups, the estimated parameters were correlated. In G1/2 tumors, all PET parameters correlated well (Table 3). In G3 tumors, PET parameters also have shown significant correlations (Table 4). Finally, PET imaging–based parameter values did not correlate with ADC_mean_ in both groups.

**Table 3:** Correlations between DWI and PET parameters in G1 and 2 tumors.

**Parameters**	**SUV_**max**_ (lbm)**	**SUL_**peak**_(SUV-lbm / Size)**	**TLG (SUV-lbm × cm^**3**^)**	**MTV (cm^**3**^)**	**ADC_**mean**_**
SUV_max_ (lbm)	-	***r*** **= 0.97** ***p*** **< 0.0001**	***r*** **= 0.63** ***p*** **< 0.0001**	***r*** **= 0.31** ***p*** **= 0.03**	*r* = −0.24 *p* = 0.09
SUL_peak_(SUV-lbm / Size)		-	***r*** **= 0.73** ***p*** **< 0.0001**	***r*** **= 0.42** ***p*** **= 0.01**	*r* = −0.27 *p* = 0.06
TLG (SUV-lbm × cm^3^)			-	***r*** **= 0.89** ***p*** **< 0.0001**	*r* = −0.21 *p* = 0.16
MTV (cm^3^)				-	*r* = −0.07 *p* = 0.59
ADC_mean_					-

*Significant correlations are highlighted in bold*.

**Table 4:** Correlations between DWI and PET parameters in G3 tumors.

**Parameters**	**SUV_**max**_ (lbm)**	**SUL_**peak**_(SUV-lbm / Size)**	**TLG (SUV-lbm × cm^**3**^)**	**MTV (cm^**3**^)**	**ADC_**mean**_**
SUV_max_ (lbm)	-	***r*** **= 0.97** ***p*** **< 0.0001**	***r*** **= 0.83** ***p*** **< 0.0001**	***r*** **= 0.47** ***p*** **= 0.049**	*r* = 0.79 *p* = 0.75
SUL_peak_(SUV-lbm / Size)		-	***r*** **= 0.86** ***p*** **< 0.0001**	***r*** **= 0.53** ***p*** **= 0.02**	*r* = 0.21 *p* = 0.93
TLG (SUV-lbm × cm^3^)			-	***r*** **= 0.83** ***p*** **< 0.0001**	*r* = −0.46 *p* = 0.86
MTV (cm^3^)				-	*r* = −0.36 *p* = 0.88
ADC_mean_					-

*Significant correlations are highlighted in bold*.

### Measured Parameters and Response

**Figure 5:**
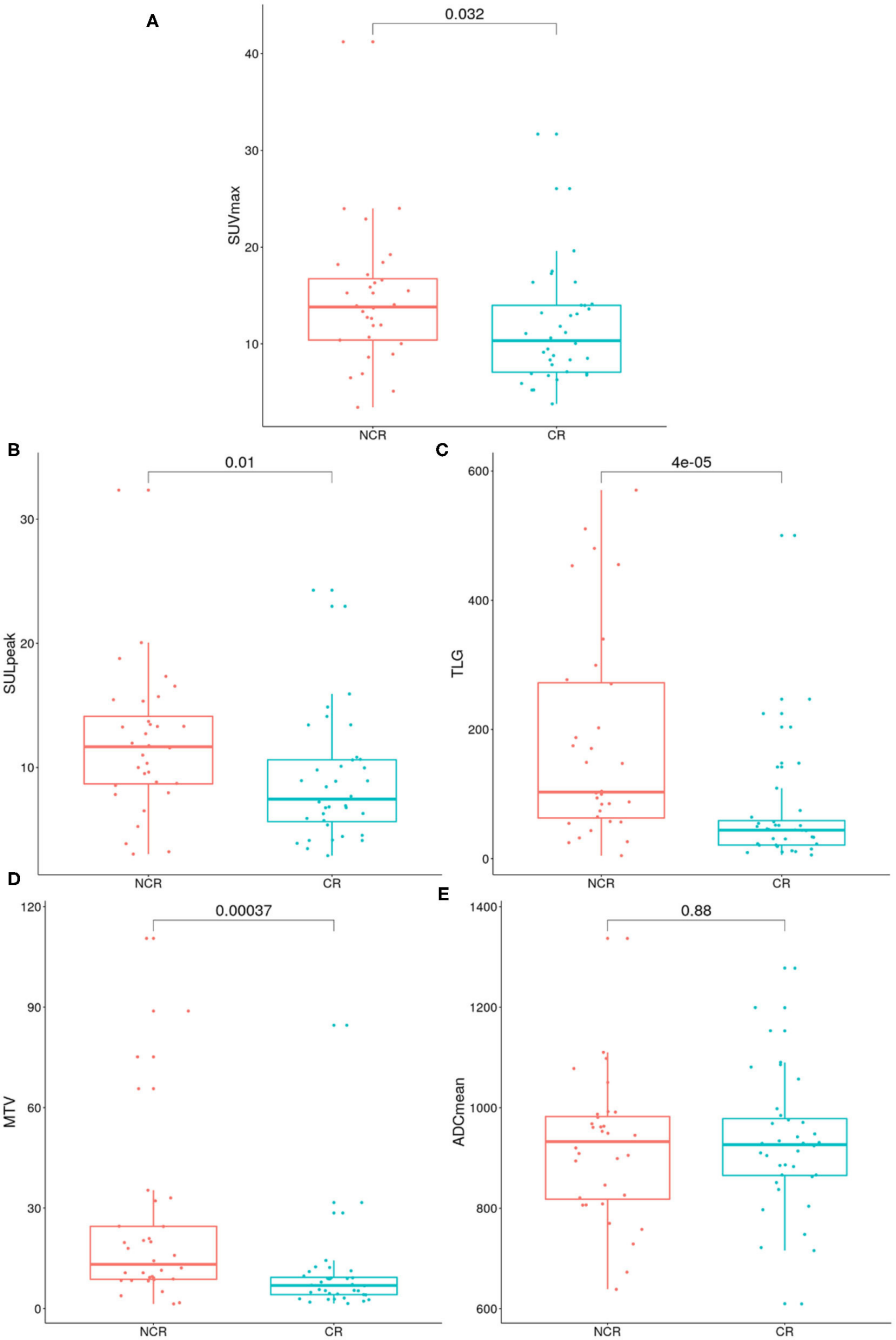
Boxplots show the distributions of maximum of standardized uptake value (SUV_max_) **(A)**, peak lean body mass corrected SUV_max_ (SUL_peak_) **(B)**, total lesion glycolysis (TLG) **(C)**, metabolic tumor volume (MTV) **(D)**, mean apparent diffusion coefficient (ADC_mean_) **(E)** parameters in the therapy response based subgroups, patients who achieve complete remission and non-complete remission. The *p*-value of the Wilcoxon rank-sum test is shown in the upper part of the plot.

According to Wilcoxon's rank-sum test, no statistically significant difference was found for the ADC_mean_ (*p* = 0.88) of patients that achieved a complete response and subjects with a viable tumor tissue after CRT. Nevertheless, SUV_max_, SUL_peak_, TLG, and MTV (*p* = 0.032, *p* = 0.01, *p* < 0.0001, *p* = 0.0004) proved to be significantly different between the two different outcome groups (Figure 5).

## Discussion

The radiotherapy of HNSCC patients based on modern complex oncological treatment is usually combined with chemotherapy and/or surgical resection (27). HNCs still have a bad overlook in the overall prognosis of the combined treatment modalities. An overall loco-regional recurrence may occur in up to 40% of locally advanced head and neck patients after the first 2 years (28). Due to the anatomical features of the head and neck region, organ preservation is important to maintain functions and to minimize aesthetic changes (29). Hoffman et al. raised some attention regarding neoadjuvant treatment strategies for tumor reduction before surgery. They also pointed out the efficacy of CRT and neoadjuvant chemotherapy followed with definitive radiotherapy for advanced HNSCC patients (30).

The study also highlighted the need to accurately predict the outcome of possible treatment options in daily clinical practice. The high mortality rate of advanced HNSCC patients and the precise cancer staging of radical resections are, therefore, essential, as both allow clinicians to select the relevant treatment strategies that could predict the prognosis of the patients (31). Hence, it is essential to identify the potential predictive indicators for these treatments.

Pretreatment ^18^F-FDG-PET/MR were evaluated for their predictive value for clinical outcomes. It is crucial to prognosticate the disease response of treatments in the pretreatment period to establish a more aggressive treatment for selected HNSCC patients (7, 32).

Overall, in this research, we focused on the combined role of DWI and PET imaging parameters for predicting tumor response to therapy in the head and neck region.

In this examination, SUV_max_, SUL_peak_ MTV, and TLG values of HNSCC patients were the predictive factors for determining response to therapy. After CRT, the risk of NCR was significantly higher in patients with high SUV_max_, SUL_peak_, MTV, and TLG values than in patients with low SUV_max_, SUL_peak_, MTV, and TLG values. Thus, the current results confirm that both TLG and MTV can add valuable information for prediction, further supporting Pak et al.'s finding, which argues that patients who have a higher risk of death and adverse events have high MTV or TLG (33). Additionally, in the current study, the non-complex (SUV_max_, SUL_peak_) parameters supported this finding as well.

The present study investigated numerous patients treated with CRT and diagnosed with histopathologically proven HNSCC. Furthermore, the study also investigated the correlations between PET and MRI-DWI parameters that were acquired simultaneously.

Via this approach, these parameters could be used to select a treatment strategy to address the higher SUV_max_, SUL_peak_, TLG, and MTV values that indicate a poorer treatment outcome. Therefore, it is worth taking the parameters suggested above into daily routine, especially the ones that significantly predict the patient outcome in daily routines to achieve more patient-tailored therapy.

Several studies found negative associations between SUV_max_ and ADC values (34, 35). However, in our study, no significant linear correlations were found between the investigated parameters. Our results are similar to the results found by Rasmussen et al. Furthermore, when we classified the patients into two different groups based on the primary tumor degree of differentiation, no significant correlations were found.

Contrary to a study by Wong et al., who reported that the ADC was a predictive factor to assess response to chemo-radiotherapy, we couldn't find a significant difference in the post-treatment ADC_mean_ between the two groups, there was no noticeable difference in the ADC values (36).

The simultaneous imaging in PET/MR provides the same bed positions and acquisition at the same time, which leads to more accurate results compared to studies that have examined them separately on individual modalities. Compared to previous studies, this study finds that both parameters (SUV_max_, SUL_peak_, MTV, and TLG) had predictive values while using the single tracer injection double imaging acquisition protocol. In this research, the SUV_max_, SUL_peak_, MTV, and TLG values were measured; thus, their predictive value was discovered very first in homogeneously treated head and neck cancer patients.

In contrast, a few limitations must be acknowledged. The 1st weak point of this study was the retrospective analysis and the single-institute implementation. Moreover, a long-term follow-up might be more accurate to determine the therapeutic response. A multicenter and prospective study with more patients could be more representative of the population. Surov et al. found that more complex PET- and DWI-based parameters proved useful to reflect several histopathological parameters (37). However, our study included only the conventional parameters, which might be one of the limitations of this research.

Despite these limitations, this report provides important contributions to the field because it is the first study to show the predictive value of SUV_max_, SUL_peak_ MTV, and TLG in patients with diagnostically confirmed HNSCC that were diagnosed with single tracer injection dual imaging acquisition. The usefulness of the ^18^F-FDG PET/MR is important; nevertheless, it has questionable added value, because ADC_mean_ has not shown significant differences between two patient groups (CR: *n* = 36 and non-CR: *n* = 32), probably due to the small number of patients (*n* = 68). In addition, this study also reported no correlations between PET and MRI-DWI based parameters. The findings suggest a need for further studies that involve more patients and more PET parameters as well as wider patient treatment modalities.

## Conclusion

Pretreatment MRI-DWI values were unable to predict therapeutic response. However, ^18^F-FDG PET parameters found to be more useful for this purpose in patients with HNSCC.

The strength of this study is the use of an MRI-DWI parameter, which includes diffusion evaluations that were collected simultaneously during PET/MR. SUV_max_, SUL_peak_ MTV, and TLG values, significantly predicted the clinical outcome; thus their inclusion in risk stratification may be of additional value for predicting patient treatment outcomes.

## Data Availability Statement

The original contributions presented in the study are included in the article/supplementary material, further inquiries can be directed to the corresponding author/s.

## Ethics Statement

The studies involving human participants were reviewed and approved by Kaposi Mor Teaching Hospital ethics committee. The participants provided written informed consent to participate in this study.

## Author Contributions

AKe and TZ designed the study. OF collected and processed DWI data. AKe conducted data collection and processing. ME and AKe did statistical analysis. ÁKo, TZ, AKe, FL, KF, and ME wrote the paper. TZ and ME review the draft and approved FDG readings. AKe segmented FDG measurements. DS, JT, and GB generated the figures. AKe, ÁKo, ZC, FK, GB, ZT, and IR discussed the results and contributed to the final form of the article. All authors have approved the final copy.

## Conflict of Interest

The authors declare that the research was conducted in the absence of any commercial or financial relationships that could be construed as a potential conflict of interest.

## Footnotes

^1^Available online at: https://www.r-project.org/

^2^Available online at: https://rpkgs.datanovia.com/ggpubr/

^3^Available online at: https://www.r-bloggers.com/easily-explore-your-data-using-the-summarytools-package/

## Abbreviations

PET/MR: Positron Emission Tomography/Magnetic Resonance Imaging
PET/CT: Positron Emission Tomography/Computed Tomography
SUV_max_: Maximum Standardized Uptake Value
SUL_peak_: Peak Lean Body Mass Corrected SUV Uptake Value
MTV: Metabolic Tumor Volume
TLG: Total Lesion Glycolysis
CRT: Chemoradiotherapy
HNSCC: Head and Neck Squamous Cell Carcinoma
FDG: Fluorodeoxyglucose
PERCIST: Positron Emission Response Criteria in Solid Tumors
CR: Complete Remission
NCR: Non-complete Remission
KWT: Kruskal–Wallis tests
AJCC: American Joint Committee on Cancer
US: Ultrasound
FOV: Field of View
DW: Diffusion-weighted
EP: Echo Planar
TR: Time of Repetition
TE: Time of Echo
TI: Time of Inversion Recovery
TSE: Turbo Spin Echo
FS: Fat Suppression
ROI: Region of Interest
VOI: Volume of Interest
^18^F-FDG: 2-Deoxy-2-[18F]fluoro-D-glucose
ADC_mean_: Average Mean Apparent Diffusion Coefficient
ADC_min_: Minimum Apparent Diffusion Coefficient
MRAC: magnetic resonance-based attenuation correction.
